# Cigarette Smoke Regulates the Competitive Interactions between NRF2 and BACH1 for Heme Oxygenase-1 Induction

**DOI:** 10.3390/ijms18112386

**Published:** 2017-11-10

**Authors:** Wen-Hsin Chang, Philip Thai, Jihao Xu, David C. Yang, Reen Wu, Ching-Hsien Chen

**Affiliations:** 1 Department of Internal Medicine, Division of Pulmonary and Critical Care Medicine and Center for Comparative Respiratory Biology and Medicine, University of California Davis, Davis, CA 95616, USA; cwh77160@gmail.com (W.-H.C.); effectorbio@gmail.com (P.T.); dvcyang@ucdavis.edu (D.C.Y.); rwu@ucdavis.edu (R.W.); 2 Institute of Molecular Medicine, National Taiwan University College of Medicine, Taipei 10002, Taiwan; 3 Division of Nephrology, Department of Internal Medicine, University of California Davis, Davis, CA 95616, USA; jihxu@ucdavis.edu; 4 Comprehensive Cancer Center, University of California Davis, Davis, CA 95616, USA

**Keywords:** smoke, *HO-1*, NRF2, gene regulation, airway epithelium

## Abstract

Cigarette smoke has been shown to trigger aberrant signaling pathways and pathophysiological processes; however, the regulatory mechanisms underlying smoke-induced gene expression remain to be established. Herein, we observed that two smoke-responsive genes, *HO-1* and *CYP1A1*, are robustly induced upon smoke by different mechanisms in human bronchial epithelia. *CYP1A1* is mediated by aryl hydrocarbon receptor signaling, while induction of *HO-1* is regulated by oxidative stress, and suppressed by *N*-acetylcysteine treatment. In light of a pivotal role of NRF2 and BACH1 in response to oxidative stress and regulation of *HO-1*, we examined if smoke-induced *HO-1* expression is modulated through the NRF2/BACH1 axis. We demonstrated that smoke causes significant nuclear translocation of NRF2, but only a slight decrease in nuclear BACH1. Knockdown of NRF2 attenuated smoke-induced *HO-1* expression while down-regulation of BACH1 had stimulatory effects on both basal and smoke-induced *HO-1* with trivial influence on NRF2 nuclear translocation. Chromatin immunoprecipitation assays showed that smoke augments promoter-specific DNA binding of NRF2 but suppresses BACH1 binding to the *HO-1* promoter ARE sites, two of which at −1.0 kb and −2.6 kb are newly identified. These results suggest that the regulation of NRF2 activator and BACH1 repressor binding to the ARE sites are critical for smoke-mediated *HO-1* induction.

## 1. Introduction

Cigarette smoke is one of the leading causes of lung diseases worldwide. Despite widespread public health policies to reduce smoking, smoking continues to be a major cause of preventable mortality and chronic diseases such as lung cancer and chronic obstructive pulmonary disease (COPD) throughout the world [1,2]. The primary target of smoke-induced oxidative injury is the epithelial cell layer lining the respiratory tract. Airway epithelial cells up-regulate several antioxidant enzymes including cytochrome p450 (*CYP1A1*) and heme oxygenase 1 (*HO-1*) to defend against oxidative stress. *CYP1A1* gene expression in airway epithelial cells is induced by aryl hydrocarbon receptor (AhR), which is activated by polycyclic aromatic hydrocarbons (PAHs) in smoke [3], while the mechanism of smoke-induced elevation of *HO-1* expression in airway epithelial cells is still incompletely elucidated.

HO-1 is a cytosolic enzyme that catalyzes the breakdown of intracellular heme into carbon monoxide (CO), iron and biliverdin [4]. Since high intracellular levels of heme cause oxidative damage to the cells [5,6], *HO-1* knockout mice or in vitro RNAi knockdown affects cell survival and leads to enhanced tissue injury from oxidants [7,8], suggesting the significance of *HO-1* and its tight regulation in oxidative stress response and protecting cells from injury or death. NRF2, also called nuclear factor (erythroid-derived 2)-like 2 (NFE2L2), and BACH1 are purportedly upstream of *HO-1*, and both belong to a class of transcription factors called the cap ‘n’ collar basic leucine zipper (CNC-BLZ) [9]. NRF2 regulates the transcription of numerous antioxidant enzymes that are important for cellular protection against oxidative stress, including nicotinamide adenine dinucleotide phosphate, quinone oxidoreductase 1, the glutamate-cysteine ligase, catalytic and modifier subunits, and thioredoxin in addition to HO-1 [10]. 

Under stress-free conditions, NRF2 levels are kept low by polyubiquitination and proteasomal degradation maintained through its binding and association with the Kelch like-ECH-associated protein 1 (KEAP1), which serves as an adaptor linking NRF2 to the Cul-E3 ubiquitin ligase [11,12]. Activation of NRF2 occurs when free radicals, by virtue of modification of key cysteine residues on the KEAP1 adaptor, alters its conformation leading to NRF2 dissociation from the Cul3 ubiquitin ligase and translocation into the nucleus [11,12]. There, it binds to DNA elements after forming heterodimers with small maf proteins [13,14]. The DNA recognition elements of the NRF2/maf heterodimers are referred to as ARE (antioxidant responsive element) or MARE (maf-associated responsive element). Less is known about BACH1 compared to NRF2. BACH1 serves as a transcriptional repressor, and in many cases antagonizes the effects of NRF2 and recognizes similar or identical DNA elements with NRF2 [15,16]. Previous studies have characterized two putative AREs, EN1 and EN2, located at the −4.0 and −9.0 kb region of the *HO-1* promoter, respectively, and both sites bind NRF2 and BACH1 [15,17,18,19,20]. However, the mechanism underlying how DNA binding of NRF2 and BACH1 is regulated in airway epithelium and its role in the context of smoke remain to be established.

In this study, we demonstrated that smoke induces *HO-1* expression in human bronchial epithelial cells, and this induction is different from smoke-induced *CYP1A1*, which is AhR dependent. Next, we elucidated that in airway epithelial cells, *HO-1* induction by smoke is governed by competition between NRF2 and BACH1 on the AREs in the *HO-1* promoter, including two previously identified EN1 and EN2 sites and two newly identified enhancer sites, NB1 and NB2, at more proximal regions that may potentially participate in the regulation of *HO-1* expression in response to cigarette smoke exposure.

## 2. Results

### 2.1. HO-1 and CYP1A1 Are Induced by Smoke Exposure through Distinct Mechanisms

**Figure 1:**
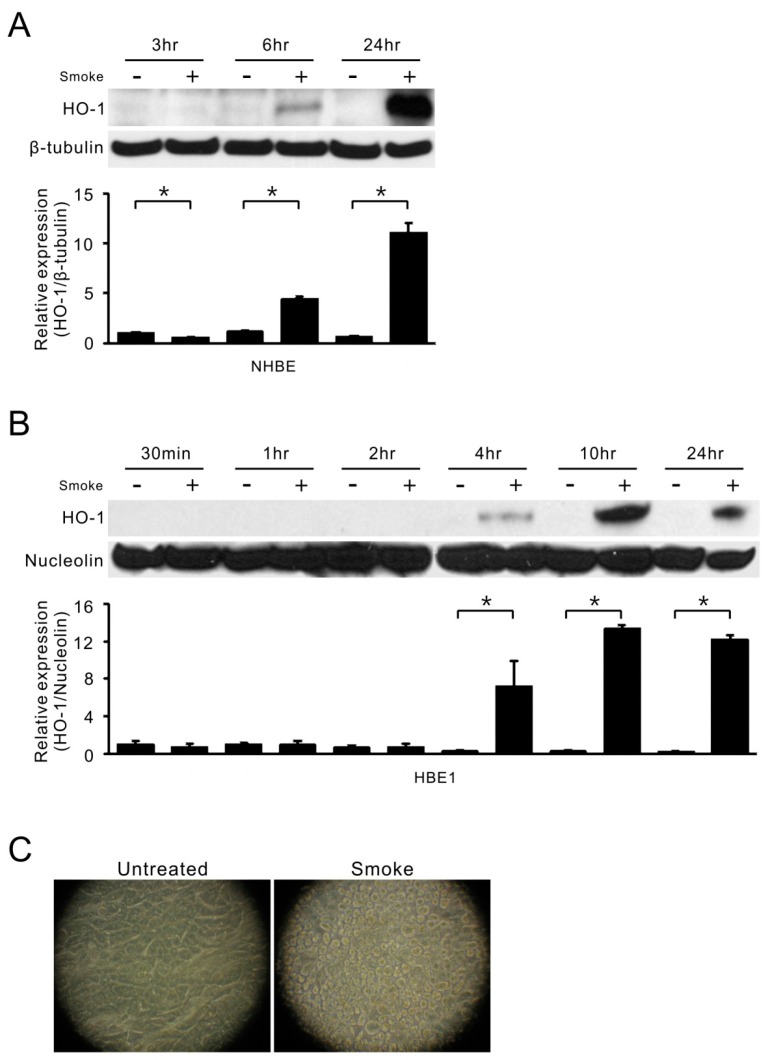
Smoke induction of *HO-1* in human airway epithelial cells. (**A**) HO-1 protein levels in NHBE cells were analyzed by Western blot assays at 3, 6 and 24 h after the start of smoke treatment (10% cigarette smoke extract) as shown in the top of the figure. β-tubulin was used to normalize the protein loading in the SDS-PAGE. Bottom, the mean results for densitometric scans of three blots from three separate experiments were expressed as fold relative to that of untreated NHBE cells at 3 h. * *p* < 0.05 (*n* = 3; mean ± S.D.); (**B**) HBE1 cells were treated with cigarette smoke and collected at indicated time points. The cell lysates were subjected to immunoblotting for HO-1 protein levels (*top*). Nucleolin was used to normalize the protein loading in the SDS-PAGE. The mean results for densitometric scans of three blots from three separate experiments are shown in the bottom panel as fold relative to that of untreated HBE1 cells at 30 min. * *p* < 0.05 (*n* = 3; mean ± S.D.); (**C**) HBE1 cells were exposed to smoke extract for 24 h and cell morphology was photographed.

Cigarette smoke is one of the major causes of oxidative stress and always triggers antioxidant activity and enzyme expression. To understand both *HO-1* and *CYP1A1* expression in response to smoke exposure, two human bronchial epithelial cells, NHBE primary cells and HBE1 cell line, were directly exposed to smoke [21]. As shown in Figure 1, the protein level of HO-1 was significantly induced, as early as 6 h in NHBE cells (Figure 1A) and 4 h in HBE1 cells (Figure 1B), after a single smoke exposure. This stimulation was time-dependent with maximum induction at 10–24 h. Figure 1C shows that cell morphology was changed after 24 h of smoke exposure, albeit there were no floating/dead cells seen in the smoke-treated group.

**Figure 2:**
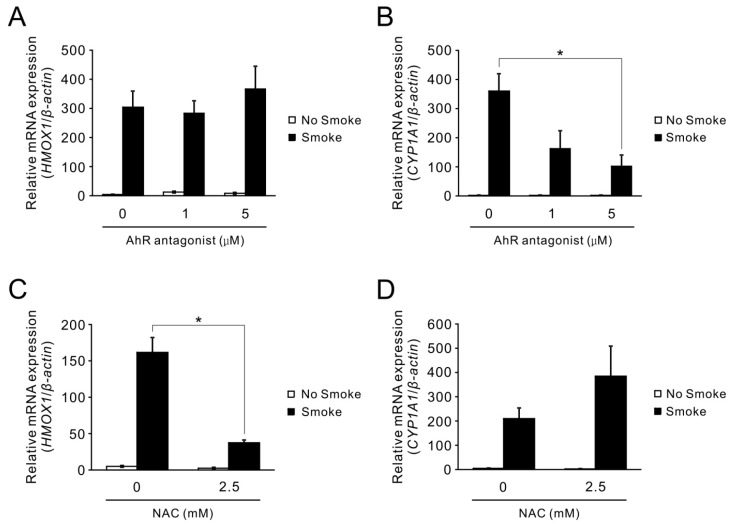
Differential mechanisms of cigarette smoke-induced *HO-1* and *CYP1A1* gene expression. (**A**,**B**) HBE1 cells were pre-treated with 1 or 5 μM AhR antagonist followed by cigarette smoke at 180 mL for 20 min and analyzed for *HO-1* (**A**) and *CYP1A1* (**B**) mRNA level by real-time quantitative reverse transcriptase polymerase chain reaction (qRT-PCR) at 24 h. * *p* <0.05 (*n* = 3; mean ± S.E.M.); (**C**,**D**) HBE1 cells were pre-treated with 2.5 mM *N*-acetylcysteine (NAC) and then exposed to smoke treatment for 24 h. The mRNA levels of *HO-1* (**C**) and *CYP1A1* (**D**) were assessed by qRT-PCR. * *p* < 0.05 (*n* = 3; mean ± S.E.M.).

We next assessed if the increased protein level of HO-1 originates from elevated mRNA levels. Additionally, the expression of another critical antioxidation-related gene, *CYP1A1*, was also examined under the same smoke treatment. Figure 2A,B shows that both *HO-1* and *CYP1A1* mRNA expression levels were notably up-regulated upon exposure to smoke. Given that *CYP1A1* is stimulated by AhR, a receptor that interacts with smoke component PAHs and leads to receptor activation [3], it was logical to investigate if a similar mechanism is applicable for smoke-induced *HO-1* expression. To test this potential mechanism, 1 or 5 μM of an AhR antagonist, 2-Methyl-2H-pyrazole-3-carboxylic acid-(2-methyl-4-*o*-tolyl-azophenyl)-amide, was added an hour prior to smoke exposure. Treatment with the AhR antagonist attenuated the smoke-induced *CYP1A1* expression as expected (Figure 2B), but this attenuation did not occur in smoke-induced *HO-1* expression (Figure 2A). Since *HO-1* induction has been shown to be associated with oxidative stress in other models, we explored whether smoke-induced *HO-1* expression occurred through a similar mechanism in airway epithelial cells. Pre-treatment of HBE1 cells with 2.5 mM of *N*-acetylcysteine (NAC), an augmenter of intracellular glutathione, attenuated smoke-induced *HO-1* (Figure 2C), but not *CYP1A1* induction (Figure 2D). This indicates that free radical species in smoke, which are scavenged by intracellular glutathione, may induce *HO-1* gene expression but are not critical for AhR-dependent *CYP1A1* induction. Thus, the induction of *HO-1* by smoke is oxidant-dependently but AhR- independently regulated.

### 2.2. Gas Phase Smoke Induces NRF2 Stabilization and Nuclear Translocation

**Figure 3:**
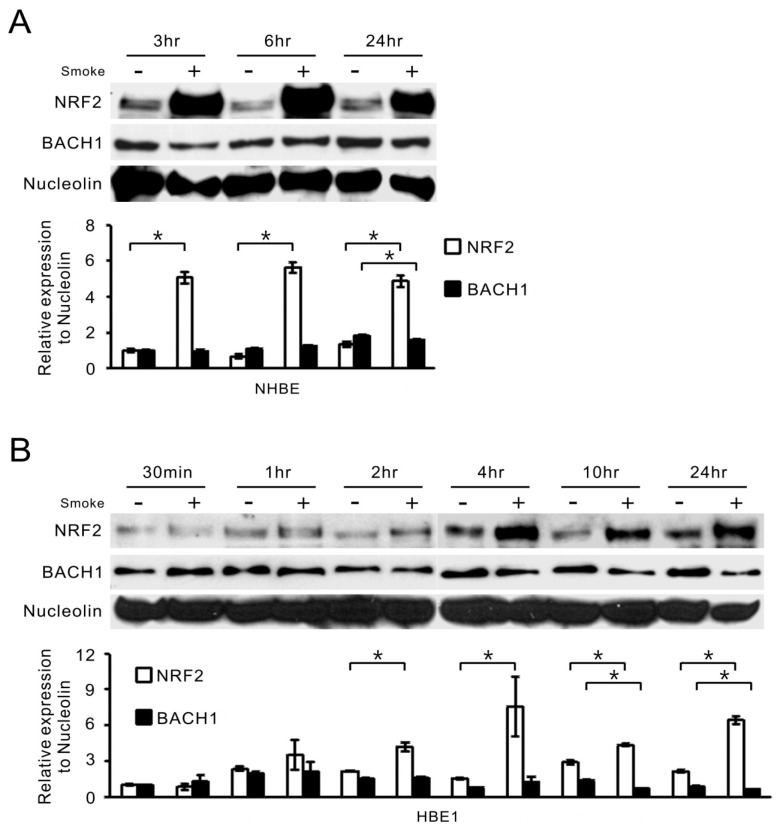
Cigarette smoke alters the nuclear levels of NRF2 and BACH1 in NHBE and HBE1 cells. (**A**,**B**) NHBE (**A**) and HBE1 (**B**) cells were collected at indicated time points after smoke exposure and extracted for nuclear proteins. The NRF2 and BACH1 protein levels in the nucleus were examined by Western blot assays (*top*). Nucleolin was used to normalize the protein loading in the SDS-PAGE gel. The mean results for densitometric scans of three blots from three separate experiments are shown in the bottom panel as fold relative to that of untreated NHBE cells at 3 h (**A**) or untreated HBE1 cells at 30 min (**B**). * *p* < 0.05 (*n* = 3; mean ± S.D.).

One of the key transcriptional factors activated in response to oxidative stress is NRF2, also responsible for *HO-1* induction [10,22]. To test whether this mechanism was also operative in smoke-exposed human airway epithelial cells, we carried out nuclear protein fractionation in NHBE and HBE1 cells after smoke exposure. The nuclear accumulations of NRF2 protein in both human NHBE (Figure 3A) and HBE1 (Figure 3B) cells were increased and persisted to the 24 h mark after cigarette smoke treatment. Of note, a 100 kDa band that shows the occurrence of polyubiquitination of NRF2 [23] was detected in smoke-treated cells, even though the predicted molecular weight of NRF2 is approximately 72 kDa. BACH1 is known to be a repressor for NRF2, so we wondered whether its nuclear level would be changed in response to smoke. Curiously, there was a much lower reduction of nuclear BACH1 levels in response to smoke compared to those of NRF2. Only at the 10 and 24 h time points could a more significant decrease in nuclear BACH1 be seen in HBE1 cells (Figure 3B), implying that if BACH1 repression is required for *HO-1* induction by smoke, it may be through a selective loss of binding to its ARE elements rather than down-regulation of its bulk nuclear protein levels. 

### 2.3. Smoke-Induced *HO-1* Expression Is Attributed to NRF2/BACH1 Regulation

**Figure 4:**
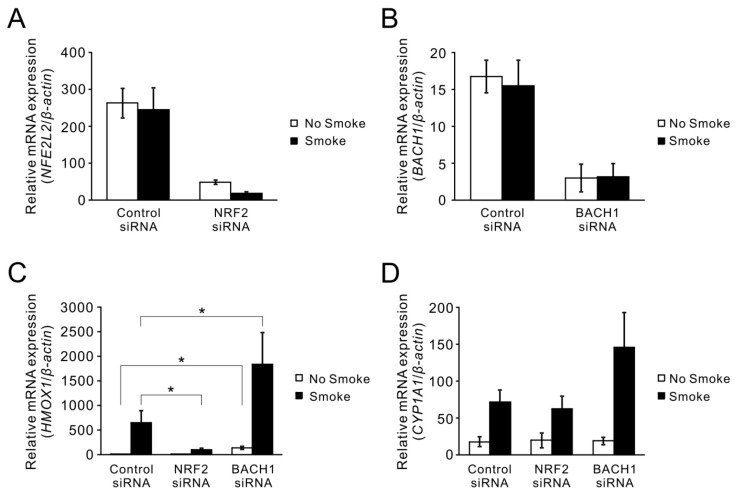
Effects of NRF2 and BACH1 on the induction of *HO-1* by cigarette smoke. HBE1 cells were transfected with control non-specific or gene-specific siRNA. After 48 h of transfection, these cells were exposed to smoke extract for additional 24 h and then subjected to qRT-PCR. (**A**,**B**) Cells were transfected with siRNA targeting NRF2 and BACH1. The knockdown efficiency of NRF2 siRNA assessed by qRT-PCR was 81% in air exposed cells and 92% in smoke exposed cells (**A**); in BACH1 siRNA treatment, the knockdown efficiency was 81% in air exposed cells and 79% in smoke exposed cells (**B**); smoke had no effect on siRNA knockdown. (**C**,**D**) Cells with siRNA knockdown of NRF2 and BACH1 followed by smoke exposure were subjected to qRT-PCR for *HO-1* (**C**) and *CYP1A1* (**D**) expression. Results are expressed as the mean across three independent experiments with error bars representing standard errors of measurements (S.E.M.). * *p* < 0.05.

Next, the functional effects of NRF2 and BACH1 on *HO-1* expression after cigarette smoke were investigated. After using siRNA to knockdown NRF2 (Figure 4A) and BACH1 (Figure 4B) expression with 70–90% efficiency, the induction of *HO-1* by cigarette smoke was abrogated by 70% under NRF2 siRNA treatment, while knockdown of BACH1 with siRNA increased the basal expression of *HO-1* and also super-induced the level of *HO-1* mRNA after smoke treatment (Figure 4C). In contrast to *HO-1* induction, smoke-induced *CYP1A1* was not affected by these siRNA treatments, although there was a slight, but not statistically significant enhancement with BACH1 siRNA treatment (Figure 4D).

**Figure 5:**
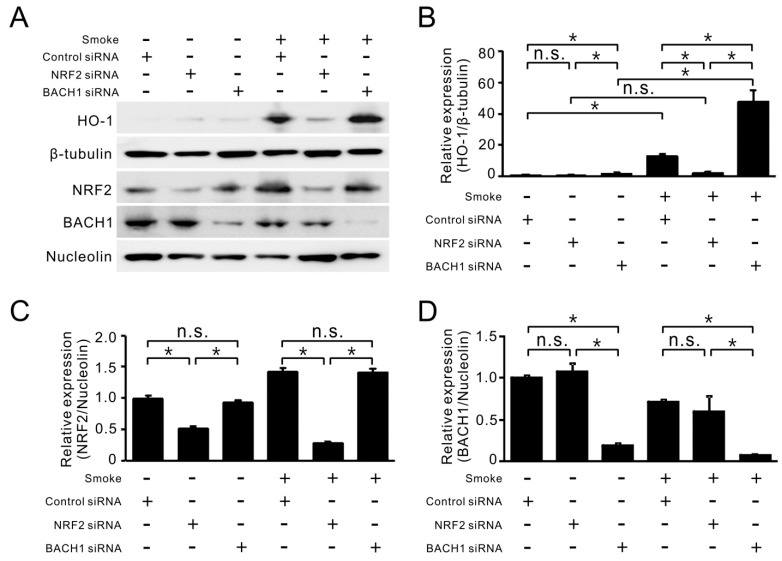

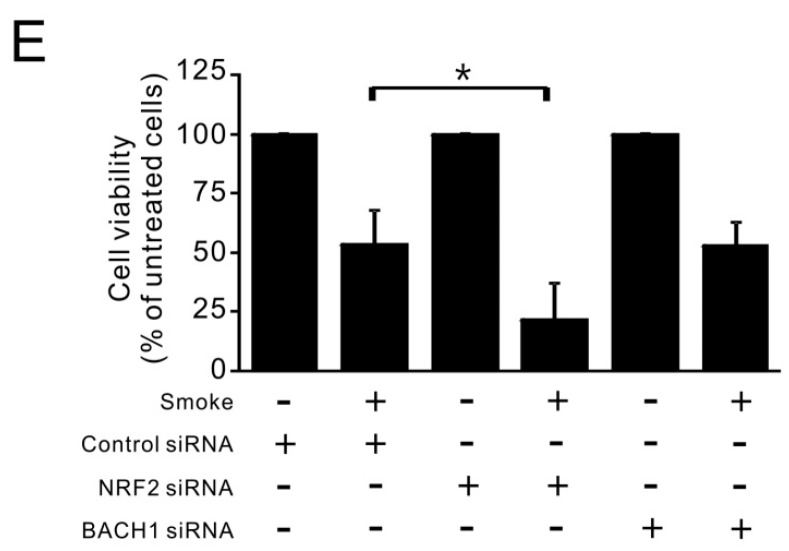
Contributions of NRF2 and BACH1 to HO-1 protein level and cell viability in response to smoke exposure. (**A**) HBE1 cells transfected with siRNA targeting NRF2 and BACH1 were subsequently exposed to cigarette smoke. After 24 h of exposure, cells were collected for protein analysis by immunoblotting. (**B**–**D**) The relative expression of HO-1/β-tubulin (**B**); NRF2/Nucleolin (**C**); and BACH1/Nucleolin (**D**) were quantified from three separate experiments as fold relative to that of untreated HBE1 cells with control siRNA. * *p* < 0.05 (*n* = 3; mean ± S.D.); (**E**) HBE1 cells with NRF2 or BACH1 siRNA transfection were exposed to cigarette smoke for 72 h and cell viability was determined by MTS assay (*n* = 3; mean ± S.D.). * *p* < 0.05.

At the protein level, NRF2 siRNA treatment effectively reduced the nuclear accumulation of NRF2 before and after smoke exposure (Figure 5A). This reduction was sufficient to block smoke-induced HO-1 protein expression (Figure 5B,C). For BACH1 siRNA treatment, it had a stimulatory effect on both the basal and smoke-induced HO-1 protein, despite having no significant effect on NRF2 nuclear accumulation (Figure 5B–D). Surprisingly, NRF2-knockdown cells were more sensitive to smoke treatment, as compared to cells receiving control siRNA (Figure 5E), suggesting the importance of NRF2 in supporting cell survival under stress. These results support the notion that the balance between NRF2 and BACH1 in the nucleus may be an important regulator for *HO-1* expression and smoke-induced cell injury. 

### 2.4. The Binding of NRF2 and BACH1 to *HO-1* Promoter Was Altered by Smoke Treatment

**Figure 6:**
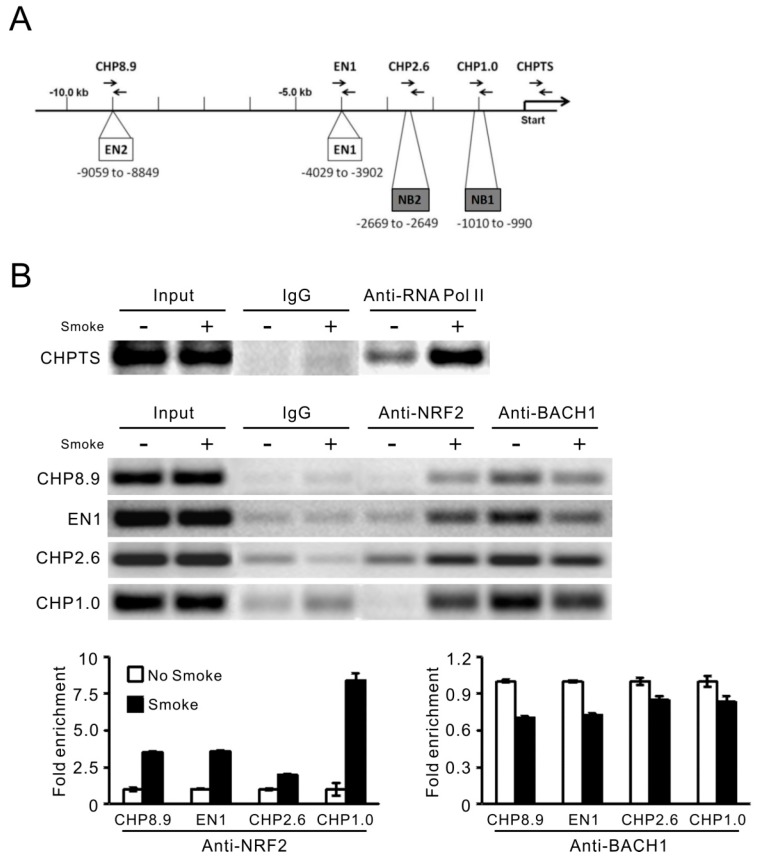
The binding sites and abilities of NRF2 and BACH1 to the *HO-1* promoter. (**A**) Diagram of putative ARE enhancer sites in the *HO-1* promoter regions for NRF2 and BACH1 binding and primers used for the ChIP assays; (**B**) ChIP assay analysis for binding of NRF2 and BACH1 to the *HO-1* gene ARE. HBE1 cells after smoke treatment were collected at 6 h, fixed in formaldehyde, and ChIP analysis was performed using IgG, NRF2 and BACH1 antibodies. The immunoprecipitated chromatin was PCR-amplified, run on agarose gel and photographed. The mean enrichment intensities are shown in the bottom panel as fold relative to that of untreated HBE1 cells (*n* = 3; mean ± S.D.).

Considering that *HO-1* expression is regulated by NRF2 and BACH1, and NRF2 and BACH1 in some cases have counteractive effects, we presumed that NRF2 and BACH1 may compete for the same ARE sites in the *HO-1* promoter region. We searched for putative binding sites by using the web-based Genomatix MatInspector, and found two previously identified −4.0 kb EN1 and −9.0 kb EN2 sites [15,18,19] and two additional sites at the −1.0 kb and −2.6 kb loci, namely NB1 and NB2, respectively (Figure 6A). Consistently, both NB1 and NB2 were also predicted to contain NRF2-binding motifs by PROMO [24] and Match™ [25], indicating the possibility of NRF2 interacting with the two regions (data not shown). To determine whether these ARE sites are involved in smoke-induced *HO-1* expression, primers covering these sites, as well as the transcriptional start site, were synthesized and used with chromatin immunoprecipitation (ChIP) analyses following immunoprecipitation with anti-RNA polymerase II, anti-NRF2 or anti-BACH1 antibodies. As shown in Figure 6B, there was a significant increase in RNA polymerase II binding to the *HO-1* transcription start site, designated by CHPTS primers, after smoke exposure. For the ARE sites, designated by the primer sets: CHP8.9 (EN2), EN1, CHP2.6 (NB2) and CHP1.0 (NB1), the anti-NRF2 ChIP assays revealed a low level of binding at EN2, EN1 and NB2 sites, and an absence of binding at the NB1 site. However, six hours after smoke exposure, elevated NRF2 binding to these ARE sites were observed. The anti-BACH1 ChIP assays demonstrated persistent binding of BACH1 to the ARE sites prior to smoke exposure. However, a decrease in binding to these promoter sites was apparent after smoke treatment, although this phenomenon was not as prominent as the increased NRF2 binding to these sites. This is consistent with the results in Figure 3, which showed much more significant changes in nuclear levels of NRF2 than BACH1 following smoke treatment.

## 3. Discussion

Smoke-induced transcriptomic profiles have been extensively studied in human airways [26,27,28,29] and mouse lungs [30]. These studies have demonstrated enhanced expression of various antioxidant and metabolic enzymes in smoke-exposed airways, while the molecular mechanism(s) underlying such induction has not been clearly demonstrated. NRF2-mediated transcriptional activation may be an important mechanism since a substantial number of NRF2 downstream genes are induced in COPD airways [26]. Because NRF2 regulates many key antioxidant enzymes, activation of this pathway by cigarette smoke likely protects cells from oxidative injury [10,22,31]. Indeed, there is evidence that *HO-1* is one such gene induced by NRF2, protecting cells from injury [32]. However, excessive activation of this pathway may also contribute to diseases such as COPD and lung cancer [26,33]. As such, understanding the precise control of NRF2 signaling will enable us to understand more about the pathogenesis of smoke-induced diseases possibly arising from the dysregulation of this pathway.

In this study, we showed that cigarette smoke triggers the nuclear translocation of NRF2 protein in airway epithelia, and this translocation and accumulation are important for induction of *HO-1* gene expression. The augmented nuclear NRF2 protein level is attributed to increased protein stability through dissociation from KEAP1 to avoid proteasomal degradation in the cytoplasm and increased import to nucleus [34]. However, the protein expression level of NRF2 was found to be diminished in patients with lung emphysema or COPD [35,36], implying increased susceptibility to oxidative stress and leading to apoptosis. The reason for NRF2 down-regulation may result from the increased level of KEAP1 [35]. An in vitro RNAi screening also indicates that inhibition of KEAP1 and some components of the ubiquitin-proteasome system has the strongest effect on NRF2 elevation [37]. Thus, targeting NRF2 inhibitors has the potential to cure or ameliorate smoke-induced diseases. Indeed, there are some NRF2 activators or inhibitors for disrupting the interaction between NRF2 and KEAP1 under development [38]. Taken together, these findings indicate that NRF2 expression is tightly regulated under normal conditions and is dysregulated in disease states.

Using siRNA and ChIP approaches, we illustrated a balance between NRF2 activation and BACH1 repression that was critical for smoke-induced *HO-1* transcriptional regulation. BACH1 is a transcriptional repressor that antagonizes the transactivation effect of NRF2 via occupying the ARE sites. Interestingly, BACH1 is also a target gene of NRF2. NRF2 binds to the ARE sites in the *BACH1* promoter regions and induces the expression of *BACH1*, forming an inhibitory feedback for NRF2-driven genes [39]. However, in our system, BACH1 mRNA and protein levels did not change much following smoke exposure, and its translocation was only slightly decreased. We thus concluded that NRF2 functions by translocation from the cytoplasm into the nucleus, while BACH1’s effects may not be due to its degradation or efflux from the nucleus, but rather a direct decrease in binding of the *HO-1* promoter following smoke exposure. The DNA binding ability of BACH1 is known to be inhibited by heme [40], and admittedly one limitation of our study is that we did not specifically test whether smoke increases the amount of intracellular heme levels. Future studies involving specific heme assays in response to cigarette smoke in our model should help clarify this mechanism.

Cigarette smoke, with its myriad amounts of reactive oxidative chemicals, induces many different signaling cascades in a variety of cells. As these experiments demonstrated, smoke can activate diverse signal transduction pathways leading to induction of different genes such as *HO-1* and *CYP1A1* in different manners. The regulation of *CYP1A1* induction in response to smoke exposure is well-documented to be triggered by PAHs-bound AhR activation [41], whereas smoke-induced *HO-1* expression is AhR-independent and its molecular mechanism remains to be determined. Our initial experiments confirmed the importance of free radicals in inducing *HO-1* and provided evidence that induction of *HO-1* involves NRF2 and BACH1. Other molecular mediators of transcriptional activation are also found to be activated by cigarette smoke and/or could regulate *HO-1* expression. HIF-1α has been shown to be an important regulator of smoke-induced *HO-1* in lung macrophages [42]. EGR-1 has been shown to be increased in COPD airways [43] and is involved in smoke-induced *HO-1* expression in lung fibroblasts [44]. SIRT1, a class III histone/protein deacetylase, was found to up-regulate *HO-1* and this is suppressed by neutrophil elastase digestion on SIRT1 [45]. NF-κB activation can be induced by smoke exposure [46], and activated NF-κB regulates *HO-1* expression in a NRF2-dependent manner [47]. Our studies were focused mainly on NRF2 and BACH1, and as suggested by functional assays involving siRNA knockdown, the relative levels of NRF2 and BACH1 are key mechanisms regulating *HO-1* induction in airway epithelial cells following smoke exposure. Consistent with this notion, siRNA knockdown of HIF-1α and the p50 subunit of NF-κB did not attenuate smoke-induced *HO-1* expression in our HBE1 or primary NHBE cells (data not included). Moreover, we also identified two new ARE sites, NB1 and NB2, on the *HO-1* promoter, more proximal and located near the transcription start site. They are likely functional but may only be important in organisms such as humans or may only respond to a specific stimulus such as cigarette smoke. Plans to investigate the functionality of these sites are currently underway.

**Figure 7:**
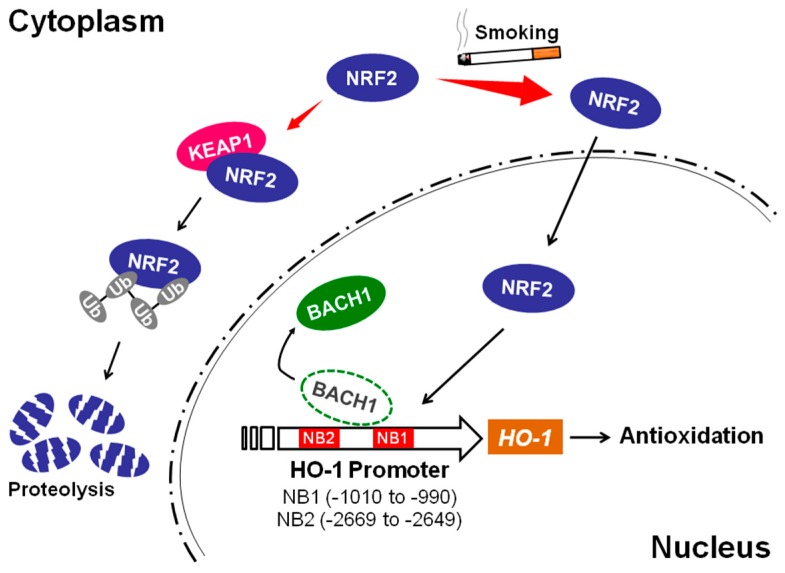
The schematic diagram of smoke-mediated *HO-1* induction. Under stress-free conditions, NRF2 is associated with KEAP1 and undergoes proteolysis. Upon exposure to cigarette smoke, NRF2 is dissociated from KEAP1 and translocated into nucleus. Nuclear NRF2 competes with BACH1 for binding to the *HO-1* promoter ARE sites, leading to *HO-1* transcription and antioxidant activity.

In summary, we have determined that the mechanism of *HO-1* induction by cigarette smoke in airway epithelial cells is dependent on NRF2 and BACH1 (Figure 7). Interestingly, we have found that in addition to the previously identified −4.0 kb and −9.0 kb enhancer binding sites, there may be two additional AREs that are more proximal, at −1.0 kb and −2.6 kb loci, that may be operative in a species or cell-type-specific manner and/or specific to cigarette smoke exposure. Further studies will help us determine whether these new promoter areas regulate *HO-1* induction and whether these sites are specifically related to smoke exposure or if they operate in a cell-type-specific manner.

## 4. Materials and Methods

### 4.1. Culturing of Human Primary Bronchial Epithelial and HBE1 Cell Line

Normal human primary bronchial epithelial (NHBE) cells were obtained from airway tissues provided from National Disease Research Interchange (NDRI) (Philadelphia, PA, USA) and UC Davis Hospital with consent as previously described [48]. The protocol for human tissue procurement was reviewed and approved by the University Human Subject Research Review Committee and consent forms for these tissues were obtained. Isolated cell pellets were suspended in Clonetics BEGM medium (Cambrex Lonza, East Rutherford, NJ, USA) with all hormones/growth factors included in the package, except the retinoic acid, and plated onto 100-mm tissue culture dishes and incubated in a 5% CO_2_ incubator at 37 °C until confluency (within 7–10 days). The cells were then trypsinized, plated onto Corning Costar Transwell (Corning, NY, USA) plates and further grown in the BEGM medium in a fully immersed condition until ~90–100% confluence. Mucociliary differentiation was then induced by switching the cells from the BEGM medium into Dulbecco’s modified Eagle’s and F12 medium at 1:1 ratio and supplemented with insulin (5 μg/mL), transferrin (5 μg/mL), epidermal growth factor (5 ng/mL), dexamethasone (0.1 nM), cholera toxin (10 ng/mL), bovine hypothalamus extract (15 μg/mL), and 30 nM all-*trans*-retinoic acid. The Transwells were maintained under an air-liquid interface (ALI) for an additional 14 days with a media change every other day until the experiments were performed. For the human HBE1 cell line (a gift from JR Yankaskas, University of North Carolina [49]), the cells were grown and maintained in the same DMEM: F12 media above except that it did not have retinoic acid, ethanolamine, MgCl_2_, and MgSO_4_.

### 4.2. Exposure of Cultured Cells to Main-Stream Smoke

Confluent cultures of both NHBE and HBE1 cells under ALI condition were directly exposed to smoke using a modified protocol from that previously described [21]. Briefly, Transwells were placed into a 36 cm × 18 cm × 3 cm aluminum tray equipped with a side port and a two-way stopcock for cigarette smoke injection. The cell culture plate was taped to the tray and a small culture dish with sterile H_2_O was placed into the tray to maintain moisture and humidity. Aluminum foil was then wrapped around the tray and file clips were used to form a tight seal. Research cigarettes (Kentucky Tobacco R&D Center, Lexington, KY, USA) were lit and fitted tightly into a plastic tube from which mainstream smoke was suctioned with a 60 mL catheter tip glass syringe. 180 mL of smoke (3 syringes full) were injected into the tray through the side port and the stopcock was closed. The cells were incubated for 3 h (for NHBE cells) or 20 min (for HBE1 cells) in a 37 °C incubator before being removed from the tray and the lid placed back over the cells. Control cells were placed in an identical container except that filtered air was injected through the side port instead of cigarette smoke. For experiments involving *N*-acetylcysteine (Sigma-Aldrich, St. Louis, MO, USA), cells were pretreated with *N*-acetylcysteine at 2.5 mM for one hour and subsequently continued for duration of treatment. The AhR antagonist, 2-Methyl-2H-pyrazole-3-carboxylic acid-(2-methyl-4-*o*-tolyl-azophenyl)-amide (EMD Biosciences, San Diego, CA, USA) was dissolved in dimethyl sulfoxide (DMSO) and added to cultures one hour prior to smoke exposure or the treatment. Mock and control siRNAs had equivalent amounts of DMSO.

### 4.3. siRNA Transfection

The siRNA for human NRF2 (GTAAGAAGCCAGATGTTAA) was previously described [10,33] and was custom synthesized by Qiagen (Valencia, CA, USA). A predesigned siRNA for BACH1 (CUUCCACUCAAGAAUCGUAtt) (s1860) and a negative control siRNA was ordered from Ambion/Applied Biosystems (Austin, TX, USA). A modified protocol for siRNA transfection based on that used by Amarzguioui [50] using Lipofectamine 2000 (Invitrogen, Carlsbad, CA, USA) was used for all the experiments. Briefly, the cells are trypsinized and counted. Lipofectamine 2000 was prepared as described by the manufacturer except that instead of using Opti-MEM (Invitrogen), we used the cells’ native serum free DMEM: F12 media (used for the HBE1 cells described above) without antibiotics. The cells were then plated with the Lipofectamine 2000 in a ratio of 200,000 cells/5 μL of Lipofectamine/100 pmol of siRNA and transfection allowed to occur overnight (16–20 h). The media was then changed and the cells placed back into their native media without antibiotics for an additional 24–48 h to allow the cells to recover from the transfection before smoke exposure experiments were performed. This method of transfection had minimal toxicity and consistently gave 70–90% knockdown of the target genes confirmed by real-time quantitative reverse transcriptase polymerase chain reaction (qRT-PCR) and immunoblotting.

### 4.4. Western Blot Analysis

For nuclear and cytosol extracts, cells were extracted using the Nuclear Extraction Kit from Panomics/Affymetrix (Santa Clara, CA, USA) according to the manufacture’s protocol. The extracts were quantified by the BioRad DC protein assay (BioRad, Hercules, CA, USA). Equal amounts of protein (30 μg/lane) were then loaded and run on a 12% SDS-PAGE gel, followed by a wet transfer to a PVDF membrane. Western blot analysis was performed as described previously [48] with anti-NRF2 (H300, Santa Cruz Biotechnology, Santa Cruz, CA, USA), anti-BACH1 (ab65026, Abcam, Cambridge, MA, USA), anti-HO-1 (ab13243, Abcam), anti-β-tubulin (Sigma-Aldrich), and anti-nucleolin (ab16940, Abcam).

### 4.5. Real-Time Quantitative Reverse Transcriptase Polymerase Chain Reaction

Quantitative RT-PCR (qRT-PCR) was carried out on an Applied Biosystems (Foster City, CA, USA) 5700 thermocycler as described previously [17]. Primers used are as followings: *HO-1* forward: 5′-AGCAACAAAGTGCAAGATTCTG-3′, *HO-1* reverse: 5′-TGTAAGGACCCATCGGAGAAG-3′, *NRF2* forward: 5′-ATTGAGCAAGTTTGGGAGGA-3′, *NRF2* reverse: 5′-AAGACACTGTAACTCAGGAATGGA-3′, *BACH1* forward: 5′-GAAGCTGCAAAGTGAAAAGGA-3′, *BACH1* reverse: 5′-TCTGCTTTGTCTCACCCAGA-3′, *β-actin* [48] forward: 5′-GCGGGAAATCGTGCGTGACATT-3′, *β-actin* reverse: 5′-GATGGAGTTGAAGGTAGTTTCGTG-3′. Relative abundance of mRNA expression after normalization with β-actin was used as the measurement of gene expression [51].

### 4.6. Chromatin Immunoprecipitation (ChIP)

To explore the interaction between transcriptional factor(s) and the putative binding site(s), chromatin immunoprecipitation was used as previously described [52]. Briefly, HBE1 cells (~10 million cells per treatment condition per antibody of immunoprecipitation) were crosslinked with 1% formaldehyde for 15 min followed by neutralization with 0.125 M glycine final concentration. The cells were then scraped off with a cell scraper and washed with ice cold PBS 3 times. These fixed cells were then re-suspended in swelling buffer (0.1 M Tris pH 7.6, 10 mM potassium acetate, 15 mM magnesium acetate, 1% Nonidet P-40 (NP-40), 1× Halt Protease Inhibitor Cocktail (Thermo Scientific), 1× PMSF (InvivoGen, Carlsbad, CA, USA), dounced 20 times, and then centrifuged to pellet the nuclei. The nuclear pellet was then re-suspended in nuclei lysis buffer (50 mM Tris-Cl, pH 8.0, 10 mM EDTA, 1% SDS, 1× Protease Inhibitor Cocktail, 1× PMSF). These re-suspended pellets were then sonicated with a Bioruptor (Diagenode) at 15 s on and 60 s off for 11 cycles. This gave sonicated DNA in the range of ~300–1000 bp for our cells. The samples were then centrifuged to remove the cell debris and the supernatant containing the chromatin was diluted 1/3 with IP dilution buffer (0.01% SDS, 1.1% Triton X-100, 1.2 mM EDTA, 16.7 mM Tris-Cl pH 8.0, 167 mM NaCl, 1× Protease Inhibitor Cocktail, 1× PMSF). This diluted lysate was then pre-cleared with Staph A cells (Pansorbin, Calbiochem), previously blocked with 1 mg/mL of salmon sperm DNA and 1 mg/mL of BSA, for 15 min at 4 °C. Aliquots (5%) of the lysates were taken for input controls. Antibodies were then added at the following amounts: 2 μg anti-NRF2 (H300, Santa Cruz, Biotechnology), 2 μg anti-BACH1 (Santa Cruz Biotechnology), 10 μg anti-RNA Pol II (Covance, Princeton, NJ, USA), and the IP incubated overnight at 4 °C. The immunoprecipitated complexes were pulled down by incubating with Staph A cells for 1 h at 4 °C. The cells were then washed 4 times with IP wash buffer (100 mM Tris-Cl pH 9.0, 500 mM LiCl, 1% NP-40, 1% Deoxycholic Acid, 1× PMSF). The DNA-protein complexes were then eluted with elution buffer (50 mM NaHCO_3_, 1% SDS). The samples were then reverse crosslinked with NaCl (final 0.2 M) and incubated overnight at 65 °C. The eluate was then incubated with RNase A at 0.1 mg/mL for 30 min and the DNA was purified with a Qiagen PCR purification kit. An aliquot of each of the purified IP DNA was then amplified by conventional PCR and the products analyzed on a 1.5% agarose gel by electrophoresis. The primers for the PCR were: *CHPTS* forward: 5′-TATGACTGCTCCTCTCCACC-3′, *CHPTS* reverse: 5′-CGCCCCGCGCTTGCCTGTC-3′, CHP2.6 forward: 5′-ATACACGCAAACTCATCTCCCCTA-3′, *CHP2.6* reverse: 5′-CCAGCCTTTATTGAGAATTTACTA-3′, *CHP1.0* forward: 5′-CTCGAACTCAAAGCAATCTTCC-3′, *CHP1.0* reverse: 5′-CTTGCTGATCGCCTATTGAATC-3′. *CHP8.9* (forward: 5′-CACGGTCCCGAGGTCTATT-3′ and reverse: 5′-TAGACCGTGACTCAGCGAAA-3′) and *EN1* (forward: 5′-CAGTGCCTCCTCAGCTTCTC-3′ and reverse: 5′-CTCGGTGGATTGCAACATTA-3′) primers were synthesized as previously described [18].

### 4.7. Cell Viability Assays

HBE1 cells were seeded into 96-well plates and cultured for the indicated treatment. Cell viability was evaluated by the MTS assays according to the manufacturer’s protocol (Promega, Madison, WI, USA). The absorbance measured at 490 nm was on a multi-well scanning spectrophotometer (Victor3; Perkin-Elmer, Boston, MA, USA).

### 4.8. Statistical Analysis

Experiments were carried out in triplicate and repeated in at least three independent passages (HBE1) or three independent NHBE cultures from different donor tissues. The numbers of experiments are stated in the figure legends as n. Standard deviation (S.D.) or standard errors of the means (S.E.M.) were calculated for all treatment groups and used for error bars seen in the figures. All experiments were analyzed by two-tailed Student’s *t*-tests or one-way ANOVA followed by multiple comparison tests for the *p* value calculation, and statistical significance were noted on the figures when *p* < 0.05.
